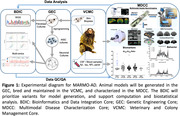# The Common Marmoset as a Preclinical Primate Model for Alzheimer’s disease

**DOI:** 10.1002/alz.087346

**Published:** 2025-01-09

**Authors:** Afonso C Silva

**Affiliations:** ^1^ University of Pittsburgh School of Medicine, Pittsburgh, PA USA

## Abstract

**Background:**

Our limited understanding of the mechanisms that trigger the emergence of Alzheimer’s disease (AD) has contributed to the lack of interventions that stop, prevent, or fully treat this disease. We believe that developing a nonhuman primate model of AD will be an essential step toward overcoming the limitations of other model systems and is crucial for investigating primate‐specific mechanisms underlying the cellular and molecular root causes of the pathogenesis and progression of AD.

**Method:**

The consortium successfully generated viable founders carrying PSEN1 mutations. in C410Y and A426P using CRISPR/Cas9 approaches, with germline transmission demonstrated in the C410Y line. Longitudinal characterization of these models, their germline offspring, and normal aging outbred marmosets is ongoing. All data and resources from this consortium will be shared with the greater AD research community.

**Result:**

The consortium successfully generated viable founders carrying PSEN1 mutations. in C410Y and A426P using CRISPR/Cas9 approaches, with germline transmission demonstrated in the C410Y line. Longitudinal characterization of these models, their germline offspring, and normal aging outbred marmosets is ongoing. All data and resources from this consortium will be shared with the greater AD research community.

**Conclusion:**

By establishing marmoset models of AD, we will be able to investigate primate‐specific cellular and molecular root causes that underlie the pathogenesis and progression of AD, overcome limitations of other model organisms, and support future translational studies to accelerate the pace of bringing therapies to patients.